# Supplementary material for: The brain is required for normal muscle and nerve patterning during early Xenopus development
Source: Nat Commun. 2017 Sep 25;8:587. doi: 10.1038/s41467-017-00597-2 (PMC5610959; doi:10.1038/s41467-017-00597-2)
Supplement: Supplementary file 2 — Supplementary Information [file 41467_2017_597_MOESM2_ESM.pdf]

Description of Supplementary Files

File Name: Peer Review File

File Name: Supplementary Information
Description: Supplementary Figures.

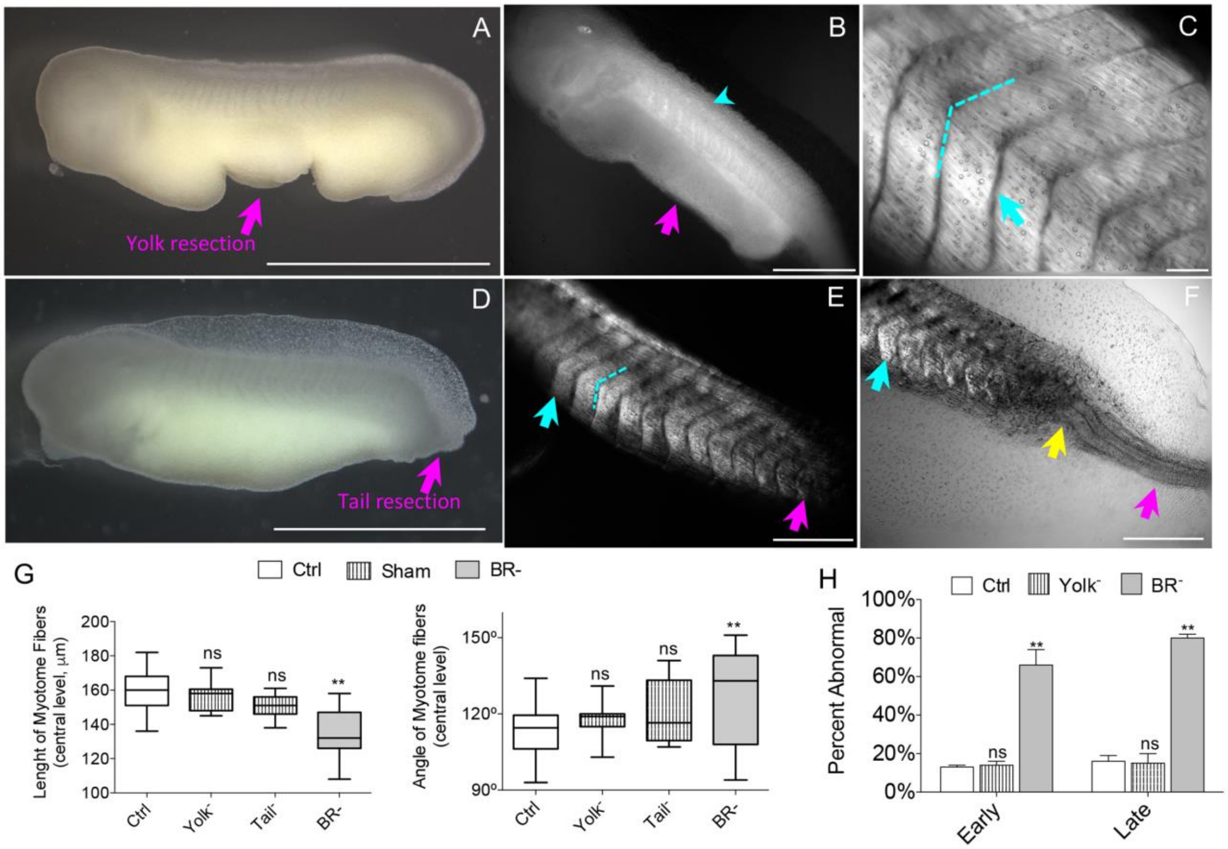

Supplementary Figure 1. Muscle phenotypes in sham or BR$^-$ embryos after either yolk or tail resection A-C. Muscle patterning in embryos where the ventral gut endoderm (magenta arrow) was partially resected at stage 25. A. Lateral view of a Yolk$^-$ embryo, three hours after injury. B. Visualization of somite organization under polarized light one week after yolk injury showed an overall normal patterning of the myotomes (turquoise head arrow). C. Three weeks after injury, Yolk$^-$ embryos exhibited both normal length/definition (turquoise arrow) and angle (turquoise dashed line) of the central myotomes. **D-F**. Muscle patterning in embryos where tailbud was resected at stage 25 (magenta arrow). . D. Lateral view of a Tail$^-$ embryo, one week after injury. E, F. Images under polarized-light (E) and combined with bright field (F) of one-week post-injury Early tailbud injury blocks the development and growth of the posterior myotomes (magenta and yellow arrows) but it does not affect the fine structure of the central myotomes (turquoise dashed line and arrow). **G**. Quantification of the mean length (*left;* one-way ANOVA, *P* <0.01) and mean angle (*right;* Kruskal-Wallis, *P* <0.05) of myotome fibers at central anatomical level of the animal body for Ctrl, Sham (both Yolk$^-$ and Tail$^-$), and BR$^-$ groups. Values are plotted as mean and s.d. of two independent replicates (*n*=50 animals per group). **H**. Quantification of the mean percentage of abnormal embryos within Ctrl, Yolk$^-$ and BR$^-$ populations at early and late developmental stages. Values are plotted as mean % and s.d. (no-pooled data from two different replicates). *n* for Yolk$^-$ embryos is 59 at early and 57 at late stages. A and D: Rostral is left and dorsal is up. Scale bar = 1 mm. B, C, E, and F: Rostral is upper left and dorsal is up. Scale bars: B, E, and F = 500 µm; C = 100 µm. G and H: statistical significance compared to Ctrl group is indicated for each group after post-hoc tests (G: Bonferroni's for length; Dunn's for angle) or z-test (H). ** *P* <0.01, ns *P* >0.05.

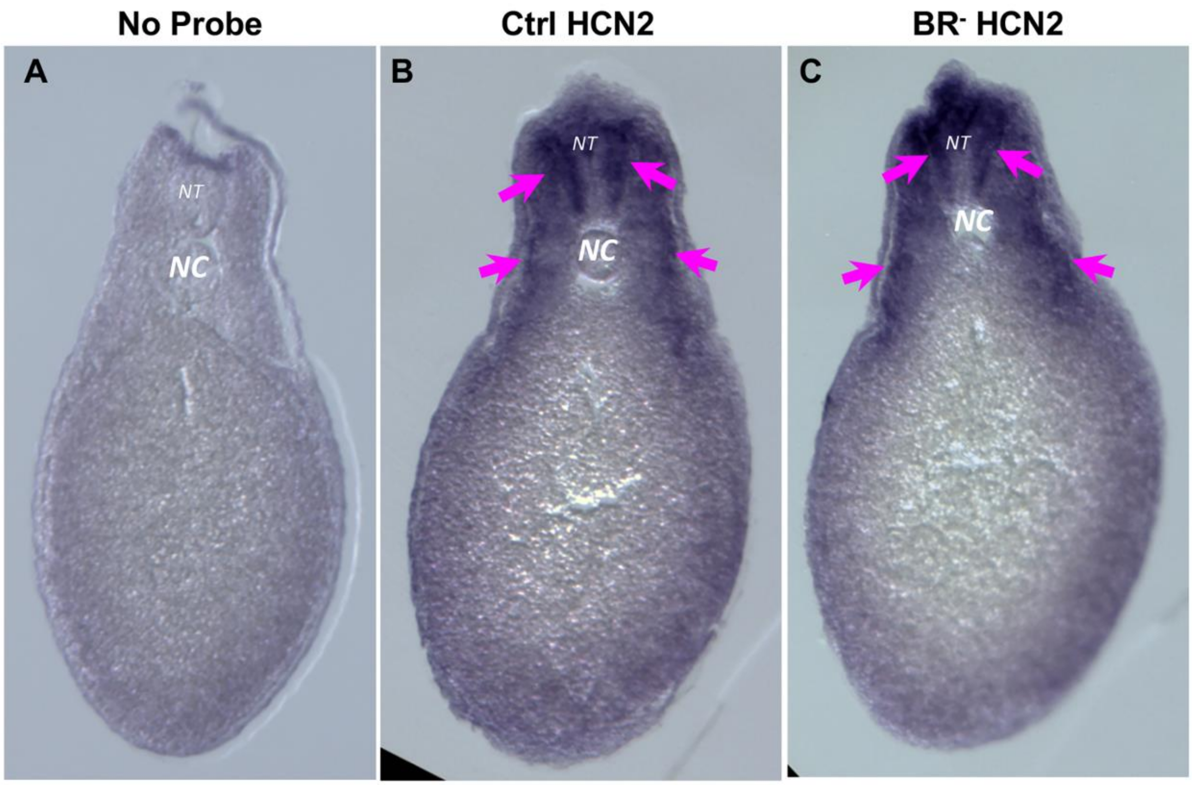

Supplementary Figure 2. *In situ* hybridization analysis of HCN2 expression in Ctrl and BR^{-} *Xenopus* embryos. *In situ* hybridization assays for HCN2 mRNA were carried out on agarose transverse sections with no probe (**A**) and anti-sense probe using stage-35 Ctrl (**B**) and BR^{-} (**C**) *Xenopus* embryos. Specific labeling was observed with HCN2 anti-sense probe in the neural tube particularly along the basal and lateral regions, and also in the perisomitic area (magenta arrows). BR^{-} embryos did not show any significant change in the pattern of signal. Dorsal is up. NT: neural tube; NC: notochord.

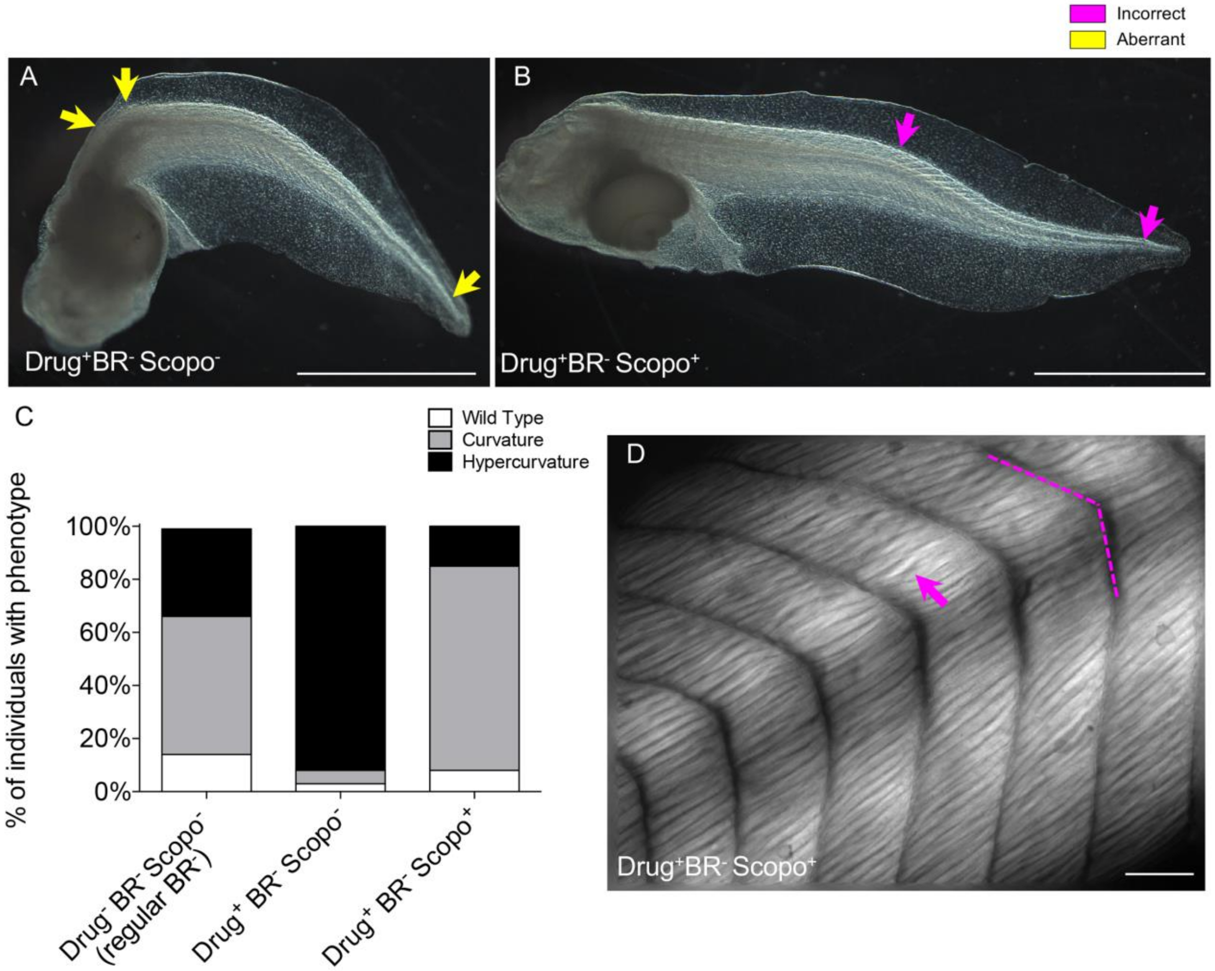

Supplementary Figure 3. Scopolamine treatment prevents drug-induced teratogenic effects on BR$^-$ embryos. **A, B**. Lateral view of stage-45 BR$^-$ embryos treated (A) with the teratogen, (Drug$^+$, (*RS*)-(tetrazol-5-yl)glycine)), but without scopolamine (Scopo) treatment (Drug$^+$Scopo$^-$) and (B) with both teratogen and scopolamine treatment (Drug$^+$Scopo$^+$). Scopolamine treatment avoided the deformities caused by the drug (such as 90-degree bending of notochord and tail, yellow arrows in A) in the BR$^-$ population, leading to the typical and less aberrant macroscopical BR$^-$ tail patterning (characterized by single lateral bending, magenta arrows in B). Rostral is to the left and dorsal is up. Magenta and yellow arrows indicate incorrect and aberrant tail modules, respectively. Scale bar = 1 mm. **C**. Analysis of the frequency of distribution of phenotypes within each BR$^-$ groups showed that scopolamine countered the effects of RS, decreasing significantly the occurrence of highly aberrant phenotypes and creating a phenotype distribution similar to the displayed in the regular BR$^-$ population (BR$^-$ without drug treatment or Drug$^-$BR$^-$Scopo$^-$) population. Data represent the pooled distribution of three replicates (*n*=75 animals per group; $X^2_{(0.05,\ 4)}$=136.3, *P*<0.01). **D**. Evaluation under polarized light of RS-BR$^-$ embryos treated with scopolamine revealed less severe alterations in the fine muscle structure than those detected in Drug$^+$BR$^-$Scopo$^-$ (see Fig. 4G for differences), resembling the typical BR$^-$ muscle phenotype [see Fig. 2D for similarities in angle (magenta dashed line) and myotome alterations (magenta arrow)]. Scale bar = 100 µm.

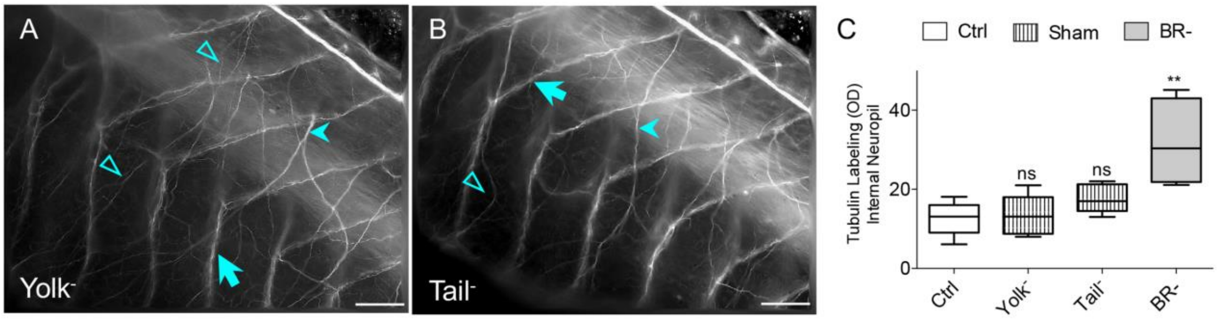

Supplementary Figure 4. Nerve phenotypes in sham or BR$^-$ embryos after either yolk or tail resection A-B. Tubulin immunofluorescence on sham embryos confirmed that the peripheral nerve architecture was not an effect derived from the early tissue-removal surgery. Three weeks after endodermal yolk mass (A) or tailbud (B) resection, embryos exhibited the typical control neural phenotype: organized commissural fibers (long turquoise arrow), structured longitudinal fibers (turquoise head arrow), and weak internal neuropil (unfilled triangles), at anatomical distance from the initial injury. **C.** Quantification of the mean OD of internal neuropil (performed on whole mount Tub-immunofluorescence) and statistical comparisons among Ctrl, Sham (either Yolk$^-$ and Tail$^-$) and BR$^-$ embryos (Kruskal-Wallis, P <0.05). Data represent the mean OD units and s.d. of two independent replicates (n=50 animals per group). P values compared to Ctrl group are indicated for each group after post-hoc Dunn's test as ** P <0.01, ns P >0.05. No significant differences were detected among the different Sham groups and Ctrl animals. A, B: Rostral is upper left and dorsal is up. Scale bar = 100 µm.

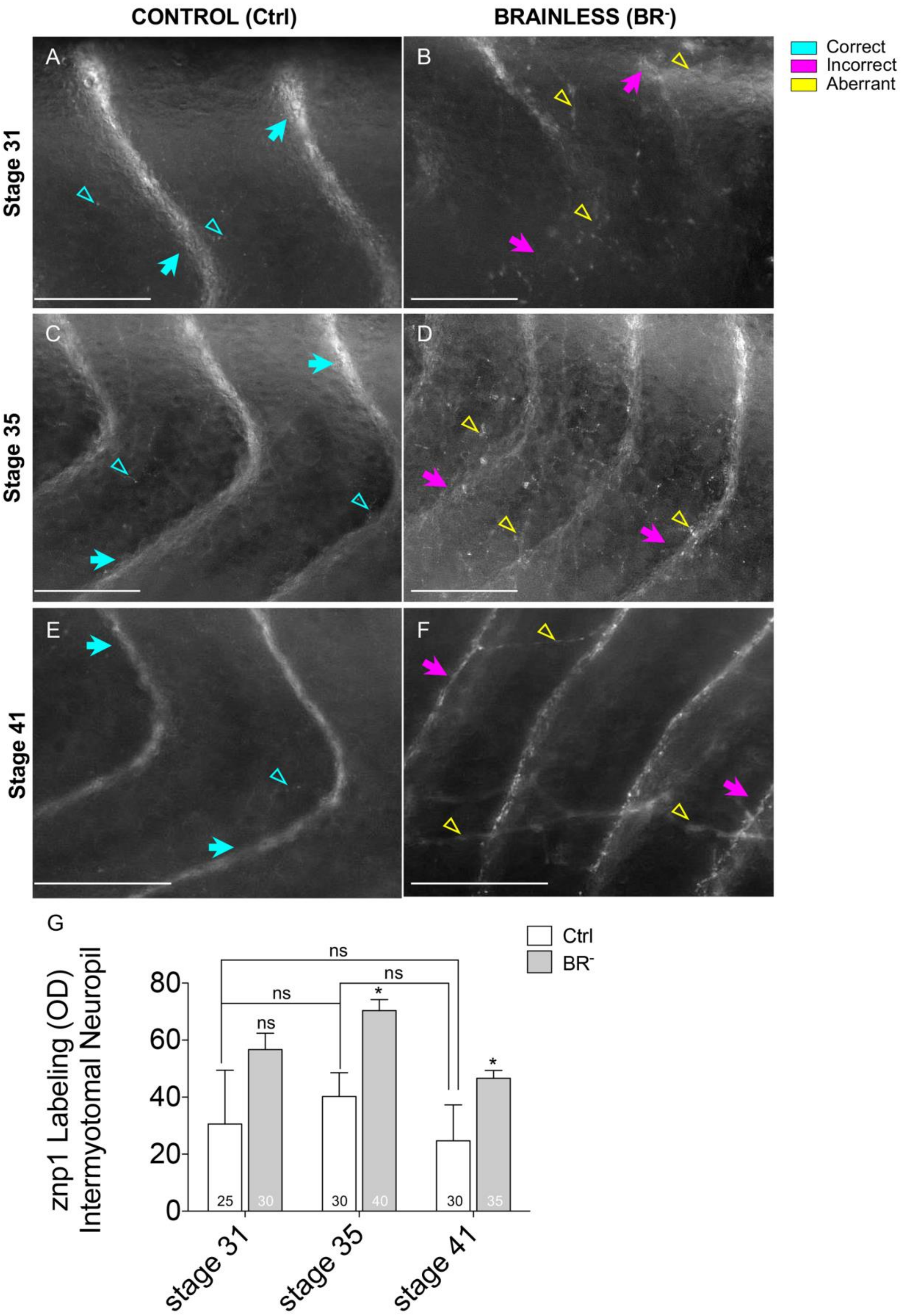

Supplementary Figure 5. Aberrant neural branching detected in BR⁻ embryos is not due to deficits in early pruning A-F. Motoneuron axonal patterning in early-staged embryos (stages 31, 35

and 41; from top to bottom, respectively) was detected by using znp1 antibody on whole-mount Ctrl (A-E) and BR^- (B-F) embryos. Motoneuron patterning errors (compare magenta arrows in B, D, and F to turquoise arrows in A, C, and E) and ectopic branching (compare yellow unfilled triangles in B, D, and F to turquoise unfilled triangles in A, C, and E) were detected from the onset and during the progression of the development in BR^- embryos. The characteristic trajectories of primary motoneuron axons at the earliest stage in Ctrl embryos (A) are not present in BR^- (B), as dorsal axons fail in turning and projecting to the midline. At stage 35, Ctrl embryos show few branches projecting off the main axons (turquoise unfilled triangles in C). This patterning is altered in BR^- embryos, with ectopic branches projecting off in an intense and disorganized manner (yellow unfilled triangles in D). A similar patterning is detected in stage-41, with a low number of nerve branches in Ctrl (E) compared to the intense axonal branching in the ventral region of the BR^- embryos (F). Rostral is right and dorsal is up. Turquoise, magenta, and yellow arrows indicate correct, incorrect, and aberrant nerve fibers, respectively. Scale bar = 100 μm. **G**. Quantification of the mean OD of internal neuropil (performed on whole mount znp1-immunofluorescncence) and statistical comparisons among Ctrl and BR^- embryos at three different early time points (two-way ANOVA, $P<0.01$ for group factor and $P<0.05$ for time factor). No significant differences were detected among Ctrl animals at different time points, indicating no significant pruning effect is identified at these stages. A value over the BR^- bar indicates statistical comparison to the respective Ctrl group. Data represent the mean OD units and s.d. of two independent replicates. Number in bars indicates *n* or number of animals analyzed for each group. *P* values after post-hoc test are indicated as * $P<0.05$, ns $P>0.05$.
